# Factors Affecting the Choice of Implant Specialists Among the Saudi Population: A Cross-Sectional Study

**DOI:** 10.7759/cureus.38419

**Published:** 2023-05-02

**Authors:** Bader Fatani, Saleh F Alrumayyan, Reema M Alsubaie, Mohammed S Alhussayen, Osama A Alharbi, Reham F Alsaleh, Afraa Al-Safadi

**Affiliations:** 1 College of Dentistry, King Saud University, Riyadh, SAU; 2 Department of Pharmacy, King Khaled University Hospital, King Saud University Medical City, Riyadh, SAU

**Keywords:** oral surgeon, periodontist, prosthodontist, factors, dental implant

## Abstract

Background

Patients are frequently exposed to misleading information about dental implants on social media or from dental marketing businesses. Patients' selection of an implant specialist for placing dental implants may vary due to several factors, including cost, social media, dentist qualification, and previous patient experience.

Objective

This study aims to assess and evaluate the factors that influence the choice of implant specialist in the Saudi population.

Materials and methods

The data were collected from 625 participants in Riyadh, Saudi Arabia, from January 2023 to April 2023. The study targeted males and females over 20 years old living in Riyadh, Saudi Arabia, and excluded those under 20 years old or not living in Riyadh, Saudi Arabia.

Results

The dentist's qualification (80.2%), dental implant cost (77.3%), and clinic or hospital (68.2%) were the most frequently agreed-upon factors for selecting an implant specialist. The study found that 13.7% of respondents disagreed or strongly disagreed that social media could influence the choice of an implant specialist. Among females, those with higher educational levels, middle-income groups, and those who had ever replaced a missing tooth, the importance of a dentist's specialty to perform a dental implant increased significantly.

Conclusion

Different factors contributed to the selection of an implant specialist among the population, with dentist qualification followed by dental implant cost being the most commonly discussed factors in the study.

## Introduction

Tooth loss is a critical oral health problem that negatively affects the population worldwide [1,2]. The prevalence of tooth loss among the adult Saudi population is reported to be high [1]. Previous studies have stated a prevalence of 69% of one or more missing teeth, with 18,640 permanent teeth extracted in Saudi Arabia in 2019 [1,2]. Dental implants have been accepted as an alternative treatment option for replacing missing teeth and have grown in the restorative field worldwide [1-4]. The advantages of a dental implant include long-term retention, improved functioning, masticatory efficiency, and improved quality of life [1,5-7]. The success of dental implants depends on their osseointegration with the osseous tissues [5]. On the other hand, osseointegration depends on the quality and quantity of bone, implant loading, and implant material used [5]. Patients are frequently exposed to misleading information about dental implants through social media or dental marketing businesses [8]. Social media marketing such as 'implants last lifelong' or 'implant forever' has promoted high expectations and unrealistic prospects among patients [9]. Reduced knowledge and awareness regarding dental implants have become a challenge, particularly in developing countries [2]. Previous studies discussed the knowledge and awareness of the population regarding dental implants [1,2,5-9]. However, no study was conducted to evaluate the factors that affect the choice of an implant specialist among the population. A previous study in Saudi Arabia reported that each dental specialty had its own treatment plan for dental implant placement, with significant knowledge observed in each specialty [10]. A patient's selection of an implant specialist for placing dental implants can demonstrate a different opinion between each patient; this can be due to several factors, such as cost, social media, dentist qualification, and previous patient experience. This study aims to assess and evaluate the factors that influence the choice of implant specialist in the Saudi population.

## Materials and methods

This research is an observational cross-sectional study with a sample size of 625 participants. Permission was obtained from the Research Ethics Committee of King Khaled University Hospital (KKUH) in Riyadh, Saudi Arabia (No. E-23-7518). The cross-sectional study design is suitable for the objective of this research. The target sample size was estimated using power analyses after consultation with a statistician. Using a confidence interval of 85%, a standard deviation of 0.5, and a margin of error of 5%, all information regarding the research questionnaire was explained, and the consent form was approved by each participant. The study was conducted from January 2023 to May 2023, targeting males and females over 20 years old who live in Riyadh, Saudi Arabia. Those under 20 years old or not living in Riyadh, Saudi Arabia, were excluded. The questionnaire contained 29 questions, which were reviewed by two specialized reviewers to ensure validity and reliability before being distributed to multiple residential areas in Riyadh, including the North, South, Middle, Eastern, and Western Riyadh cities. Our study variables are nationality, gender, age, and socioeconomic level (educational level, area of residency, and income). The sample was collected through simple random sampling using electronic questionnaires (Google Forms) distributed via social media platforms. The type of clinic or hospital was also investigated among the participants in order to determine whether it could affect the choice of implant specialist. The data were analyzed using RStudio (R version 4.2.2, RStudio, Boston, MA), with frequencies and percentages used to present categorical variables. A multiple-response analysis was applied to variables with multiple selections. Differences between demographic groups were assessed using Pearson's Chi-squared test or Fisher's exact test whenever appropriate, with a p-value of <0.05 indicating statistical significance.

## Results

Demographic characteristics of the respondents

Initially, a total of 625 responses were collected. However, 27 responses were excluded due to the lack of data on the primary outcome variables. Therefore, 598 responses were analyzed. Approximately half of the respondents were male (52.0%), aged 20 to 29 years (48.8%), and had a monthly income of <5000 SAR (44.6%). About one-third of them were residing in the North region of Riyadh (38.6%) and had obtained a post-graduate degree (31.1%). In general, 45.7% of the participants had at least one missing tooth, and 43.6% of them had replaced their missing teeth. Interestingly, 69.7% of the respondents thought replacing missing teeth was very important (Table 1).

**Table 1:** Demographic characteristics of the respondents.

Parameter	Category	Overall, N = 598	Ever heard about dental implants		p-value
			No, N = 32	Yes, N = 566	
Gender	Male	311 (52.0%)	18 (5.8%)	293 (94.2%)	0.621
	Female	287 (48.0%)	14 (4.9%)	273 (95.1%)	
Age	20–29	292 (48.8%)	11 (3.8%)	281 (96.2%)	0.311
	30–39	129 (21.6%)	9 (7.0%)	120 (93.0%)	
	40–49	94 (15.7%)	5 (5.3%)	89 (94.7%)	
	50–59	55 (9.2%)	5 (9.1%)	50 (90.9%)	
	>60	28 (4.7%)	2 (7.1%)	26 (92.9%)	
Monthly income (SAR)	<5000	267 (44.6%)	12 (4.5%)	255 (95.5%)	0.014
	5000–10,000	112 (18.7%)	7 (6.2%)	105 (93.8%)	
	10,000–20,000	116 (19.4%)	3 (2.6%)	113 (97.4%)	
	20,000–30,000	56 (9.4%)	4 (7.1%)	52 (92.9%)	
	30,000–40,000	28 (4.7%)	1 (3.6%)	27 (96.4%)	
	>40,000	19 (3.2%)	5 (26.3%)	14 (73.7%)	
Area of residency	North of Riyadh	231 (38.6%)	9 (3.9%)	222 (96.1%)	0.284
	South of Riyadh	50 (8.4%)	5 (10.0%)	45 (90.0%)	
	Eastern of Riyadh	162 (27.1%)	9 (5.6%)	153 (94.4%)	
	Western of Riyadh	98 (16.4%)	4 (4.1%)	94 (95.9%)	
	Middle of Riyadh	57 (9.5%)	5 (8.8%)	52 (91.2%)	
Educational level	Illiterate	47 (7.9%)	5 (10.6%)	42 (89.4%)	0.216
	Primary school	6 (1.0%)	1 (16.7%)	5 (83.3%)	
	Middle school	5 (0.8%)	0 (0.0%)	5 (100.0%)	
	High school	171 (28.6%)	9 (5.3%)	162 (94.7%)	
	Graduate/diploma	183 (30.6%)	6 (3.3%)	177 (96.7%)	
	Post-graduate	186 (31.1%)	11 (5.9%)	175 (94.1%)	
Have any missing teeth	No	325 (54.3%)	19 (5.8%)	306 (94.2%)	0.557
	Yes	273 (45.7%)	13 (4.8%)	260 (95.2%)	
Have ever replaced the missing teeth*	No	154 (56.4%)	9 (5.8%)	145 (94.2%)	0.339
	Yes	119 (43.6%)	4 (3.4%)	115 (96.6%)	
Think that the replacement of missing teeth is important	Not important at all	11 (1.8%)	5 (45.5%)	6 (54.5%)	<0.001
	Somewhat important	170 (28.4%)	16 (9.4%)	154 (90.6%)	
	Very important	417 (69.7%)	11 (2.6%)	406 (97.4%)	

Awareness and knowledge regarding dental implants

Importantly, the majority of respondents (94.6%) had never heard of dental implants. Awareness levels were significantly lower among participants with the highest monthly income (73.7% with >40,000 SAR, 95.5% with <5000 SAR, 93.8% with an income between 5000 and 10000 SAR, 97.4% with an income between 10,000 and 20,000 SAR, 92.9% with an income between 20,000 and 30,000 SAR, and 96.4% with an income between 30,000 and 40,000, p = 0.014) and those who thought that missing teeth replacement is not important at all (54.5% vs 90.6% among those who perceived that teeth replacement is somewhat important and 97.4% among those who perceived that teeth replacement is very important, p < 0.001) (Table 1).

**Figure 1:**
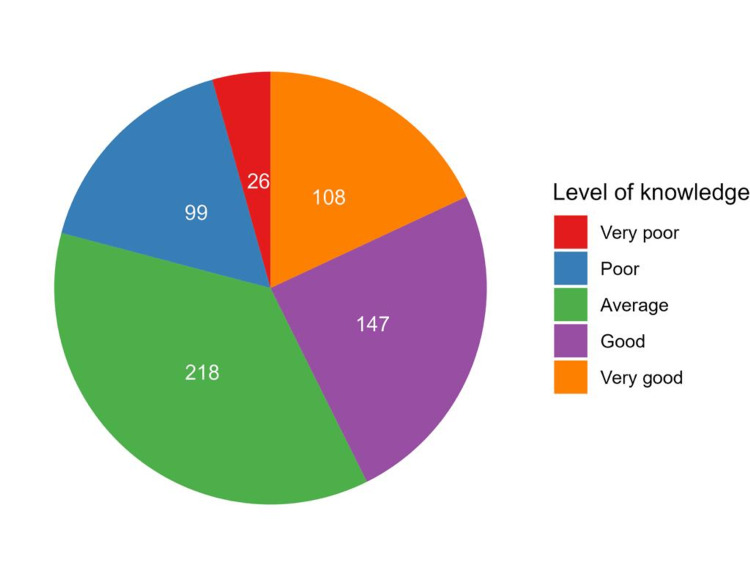
The number of participants with different categories of self-reported knowledge regarding dental implants.

Knowledge levels regarding implants were self-perceived as very poor or poor among 125 participants (20.9%), average among 218 (36.5%), and good or very good among 255 (42.6%) (Figure 1).

**Figure 2:**
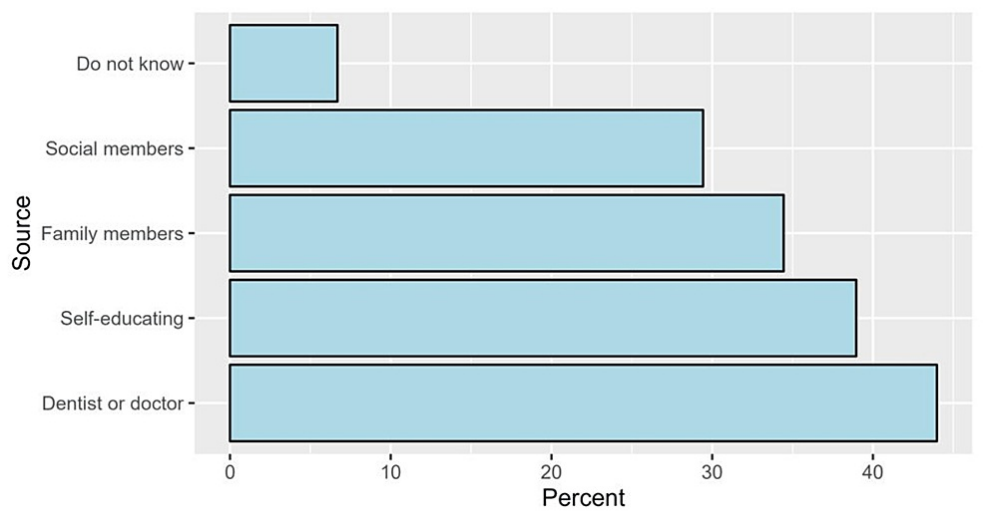
The proportions of sources of knowledge regarding dental implants.

Regarding the source of knowledge regarding implants, the most common sources were dentists or doctors (44.0%), self-education (39.0%), and family members (34.4%). Social members were (29%), and those who did not know were (7%) (Figure 2).

While 69.6% of the participants described a dental implant as a screw, only 4.7% of them had never heard about it, and 23.9% heard about it but could not explain it. More than one-third of the respondents (38.1%) thought that an implant lasts for a lifetime, and more than half of them perceived that the implants are anchored in the jawbone (59.7%) (Table 2).

**Table 2:** Knowledge about and attitudes and barriers towards dental implants.

Parameter	Category	N (%)
How would you describe a dental implant?	Never heard about it	28 (4.7%)
	Heard about it, but cannot explain	143 (23.9%)
	Piece of metal	109 (18.2%)
	Screw	416 (69.6%)
How long do you think an implant lasts?	Not sure	136 (22.7%)
	5–10 years	82 (13.7%)
	10 years	43 (7.2%)
	>10 years	109 (18.2%)
	For a lifetime	228 (38.1%)
Where in the jaw do you think implants are anchored?	I do not know	113 (18.9%)
	In neighboring teeth	35 (5.9%)
	In the gum	146 (24.4%)
	In the jawbone	357 (59.7%)
	Other	7 (1.2%)
In your view, up to which amount you need to pay for an implant?	I do not know	120 (20.1%)
	<1900 SAR	138 (23.1%)
	1900–3800 SAR	145 (24.2%)
	3800–5600 SAR	88 (14.7%)
	5600–7500 SAR	48 (8.0%)
	7500–9400 SAR	32 (5.4%)
	>9400 SAR	27 (4.5%)
Are you aware of medical problems that may interfere/lower the success rate of dental implant?	No	368 (61.5%)
	Yes	230 (38.5%)
If yes, what are the medical conditions contributing to the failure of an implant?*	I do not know	17 (7.4%)
	Gingival inflammation	164 (71.3%)
	Cancer	67 (29.1%)
	Diabetes	149 (64.8%)
	Cardiac disease	64 (27.8%)
	Bone disease	119 (51.7%)
What is the reason for not considering dental implant therapy?	Longer treatment time	121 (20.2%)
	Lack of knowledge as not given information from the dentist	144 (24.1%)
	Lack of understanding of the nature of the procedure	166 (27.8%)
	High cost	451 (75.4%)
	Fear from surgery	315 (52.7%)
	Perceived no need to replace teeth	96 (16.1%)

Attitudes and barriers towards dental implants

In general, 38.5% of the respondents indicated that they were aware of medical problems that might interfere with or reduce the success rate of dental implants; of them, the most common medical interfering factors were gingival inflammation (71.3%), diabetes (64.8%), and bone disease (51.7%). The most frequently reported barriers to considering dental implant therapy were the high cost (75.4%) and fear of surgery (52.7%) (Table 2).

Factors affecting the choice of implant specialists

**Figure 3:**
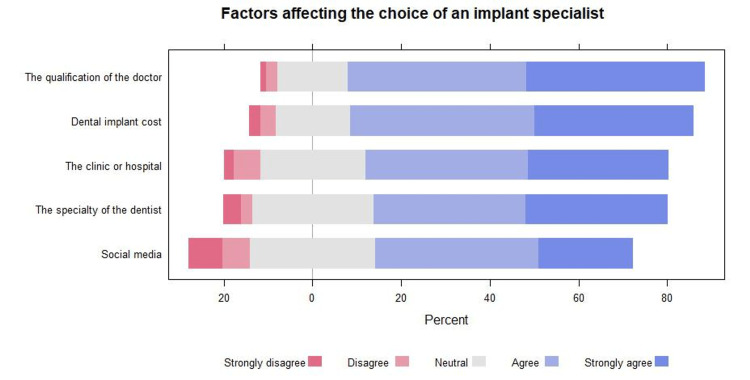
Factors affecting the choice of an implant specialist.

The most common factors on which the participants agreed or strongly agreed to select an implant specialist were the dentist's qualification (80.2%), dental implant cost (77.3%), and the clinic or hospital (68.2%). Interestingly, 13.7% of the respondents disagreed or strongly disagreed that social media could influence the choice of an implant specialist (Figure 3).

Based on participants' perceptions, the most preferred dentists to perform a dental implant were periodontal surgeons (32.1%) and prosthodontists (24.6%). A dental implant specialist should have experience in both surgery and prosthodontics (72.6%) (Table 3).

**Table 3:** Participants' responses regarding the preferred specialty and professional experience of dentists who would perform a dental implant.

Parameter	Category	N (%)
From your point of view, the specialty of the doctor you prefer to do dental implants is?	I do not care about the specialty of the doctor	78 (13.0%)
	Oral and maxillofacial surgeon	137 (22.9%)
	Prosthodontics	147 (24.6%)
	Periodontal surgeon	192 (32.1%)
	Other	44 (7.4%)
From your point of view, a dental implant specialist should have experience in?	Surgery	126 (21.1%)
	Prosthodontics	38 (6.4%)
	All of the above	434 (72.6%)

Demographic differences in the perceived factors that affect the choice of implant specialists

The proportions of participants who agreed with the importance of a dentist's specialty (prosthodontics, periodontics, or oral surgery) to perform a dental implant increased significantly among females (p = 0.005), among those with higher educational levels (p = 0.035), middle-income groups (p = 0.016), as well as among those who had ever replaced a missing tooth (p = 0.004) and those who thought that replacing a missing tooth was important (p < 0.001). The proportions of respondents who agreed or strongly agreed about the qualification of a dentist (prof, consultant, or specialist) were significantly higher among participants of the middle-income groups (p = 0.009) and thought that the replacement of missing teeth was important (p = 0.006) (Table 4).

**Table 4:** Factors associated with participants’ acceptance of the importance of the dentist's specialty and qualification in the selection of dental specialists for dental implants.

Parameter	Category	The specialty of the practicing dentist				Doctor's qualification			
		Agree or strongly agree, N = 396	Neutral, N = 164	Disagree or strongly disagree, N = 38	p-value	Agree or strongly agree, N = 480	Neutral, N = 96	Disagree or strongly disagree, N = 22	p-value
Gender	Male	190 (61.1%)	103 (33.1%)	18 (5.8%)	0.005	252 (81.0%)	52 (16.7%)	7 (2.3%)	0.163
	Female	206 (71.8%)	61 (21.3%)	20 (7.0%)		228 (79.4%)	44 (15.3%)	15 (5.2%)	
Age	20–29	183 (62.7%)	93 (31.8%)	16 (5.5%)	0.391	224 (76.7%)	57 (19.5%)	11 (3.8%)	0.540
	30–39	90 (69.8%)	31 (24.0%)	8 (6.2%)		105 (81.4%)	18 (14.0%)	6 (4.7%)	
	40–49	63 (67.0%)	24 (25.5%)	7 (7.4%)		79 (84.0%)	13 (13.8%)	2 (2.1%)	
	50–59	41 (74.5%)	9 (16.4%)	5 (9.1%)		47 (85.5%)	6 (10.9%)	2 (3.6%)	
	>60	19 (67.9%)	7 (25.0%)	2 (7.1%)		25 (89.3%)	2 (7.1%)	1 (3.6%)	
Monthly income	<5000	172 (64.4%)	82 (30.7%)	13 (4.9%)	0.016	210 (78.7%)	48 (18.0%)	9 (3.4%)	0.009
	5000–10,000	65 (58.0%)	38 (33.9%)	9 (8.0%)		89 (79.5%)	22 (19.6%)	1 (0.9%)	
	10,000–20,000	82 (70.7%)	28 (24.1%)	6 (5.2%)		99 (85.3%)	14 (12.1%)	3 (2.6%)	
	20,000–30,000	44 (78.6%)	9 (16.1%)	3 (5.4%)		48 (85.7%)	4 (7.1%)	4 (7.1%)	
	30,000–40,000	22 (78.6%)	4 (14.3%)	2 (7.1%)		23 (82.1%)	4 (14.3%)	1 (3.6%)	
	>40,000	11 (57.9%)	3 (15.8%)	5 (26.3%)		11 (57.9%)	4 (21.1%)	4 (21.1%)	
Area of residency	North of Riyadh	153 (66.2%)	64 (27.7%)	14 (6.1%)	0.940	181 (78.4%)	42 (18.2%)	8 (3.5%)	0.052
	South of Riyadh	34 (68.0%)	12 (24.0%)	4 (8.0%)		42 (84.0%)	7 (14.0%)	1 (2.0%)	
	Eastern of Riyadh	103 (63.6%)	50 (30.9%)	9 (5.6%)		142 (87.7%)	14 (8.6%)	6 (3.7%)	
	Western of Riyadh	67 (68.4%)	25 (25.5%)	6 (6.1%)		76 (77.6%)	19 (19.4%)	3 (3.1%)	
	Middle of Riyadh	39 (68.4%)	13 (22.8%)	5 (8.8%)		39 (68.4%)	14 (24.6%)	4 (7.0%)	
Educational level	Illiterate	21 (44.7%)	19 (40.4%)	7 (14.9%)	0.035	37 (78.7%)	8 (17.0%)	2 (4.3%)	0.850
	Primary school	4 (66.7%)	1 (16.7%)	1 (16.7%)		6 (100.0%)	0 (0.0%)	0 (0.0%)	
	Middle school	3 (60.0%)	2 (40.0%)	0 (0.0%)		4 (80.0%)	1 (20.0%)	0 (0.0%)	
	High school	113 (66.1%)	46 (26.9%)	12 (7.0%)		131 (76.6%)	30 (17.5%)	10 (5.8%)	
	Graduate/diploma	119 (65.0%)	53 (29.0%)	11 (6.0%)		151 (82.5%)	27 (14.8%)	5 (2.7%)	
	Post-graduate	136 (73.1%)	43 (23.1%)	7 (3.8%)		151 (81.2%)	30 (16.1%)	5 (2.7%)	
Do you have any missing teeth?	No	202 (62.2%)	99 (30.5%)	24 (7.4%)	0.072	252 (77.5%)	62 (19.1%)	11 (3.4%)	0.086
	Yes	194 (71.1%)	65 (23.8%)	14 (5.1%)		228 (83.5%)	34 (12.5%)	11 (4.0%)	
Have you ever replaced your missing teeth?	No	296 (63.1%)	142 (30.3%)	31 (6.6%)	0.004	368 (78.5%)	83 (17.7%)	18 (3.8%)	0.090
	Yes	100 (77.5%)	22 (17.1%)	7 (5.4%)		112 (86.8%)	13 (10.1%)	4 (3.1%)	
Do you think the replacement of missing teeth is important?	Not important at all	5 (45.5%)	3 (27.3%)	3 (27.3%)	<0.001	5 (45.5%)	3 (27.3%)	3 (27.3%)	0.006
	Somewhat important	99 (58.2%)	60 (35.3%)	11 (6.5%)		144 (84.7%)	22 (12.9%)	4 (2.4%)	
	Very important	292 (70.0%)	101 (24.2%)	24 (5.8%)		331 (79.4%)	71 (17.0%)	15 (3.6%)	

A significantly lower proportion of participants with the highest monthly income agreed or strongly agreed that cost plays a role in the selection of dental specialists (p = 0.006). The proportions of agreed or strongly agreed participants about the type of clinic or hospital played differed significantly based on educational level (p = 0.044) and thinking that the replacement of missing teeth is important (p < 0.001) (Table 5).

**Table 5:** Factors associated with participants’ acceptance of the importance of cost and the type of clinic or hospital in the selection of dental specialists for dental implants.

Parameter	Category	Cost				Clinic/hospital			
		Agree or strongly agree, N = 462	Neutral, N = 102	Disagree or strongly disagree, N = 34	p-value	Agree or strongly agree, N = 408	Neutral, N = 142	Disagree or strongly disagree, N = 48	p-value
Gender	Male	235 (75.6%)	53 (17.0%)	23 (7.4%)	0.158	202 (65.0%)	83 (26.7%)	26 (8.4%)	0.179
	Female	227 (79.1%)	49 (17.1%)	11 (3.8%)		206 (71.8%)	59 (20.6%)	22 (7.7%)	
Age	20–29	220 (75.3%)	56 (19.2%)	16 (5.5%)	0.291	205 (70.2%)	67 (22.9%)	20 (6.8%)	0.529
	30–39	99 (76.7%)	23 (17.8%)	7 (5.4%)		87 (67.4%)	33 (25.6%)	9 (7.0%)	
	40–49	79 (84.0%)	10 (10.6%)	5 (5.3%)		61 (64.9%)	25 (26.6%)	8 (8.5%)	
	50–59	46 (83.6%)	7 (12.7%)	2 (3.6%)		39 (70.9%)	9 (16.4%)	7 (12.7%)	
	>60	18 (64.3%)	6 (21.4%)	4 (14.3%)		16 (57.1%)	8 (28.6%)	4 (14.3%)	
Monthly income	<5000	203 (76.0%)	51 (19.1%)	13 (4.9%)	0.006	191 (71.5%)	56 (21.0%)	20 (7.5%)	0.432
	5000–10,000	91 (81.2%)	17 (15.2%)	4 (3.6%)		71 (63.4%)	33 (29.5%)	8 (7.1%)	
	10,000–20,000	94 (81.0%)	15 (12.9%)	7 (6.0%)		76 (65.5%)	31 (26.7%)	9 (7.8%)	
	20,000–30,000	47 (83.9%)	6 (10.7%)	3 (5.4%)		39 (69.6%)	13 (23.2%)	4 (7.1%)	
	30,000–40,000	19 (67.9%)	8 (28.6%)	1 (3.6%)		21 (75.0%)	4 (14.3%)	3 (10.7%)	
	>40,000	8 (42.1%)	5 (26.3%)	6 (31.6%)		10 (52.6%)	5 (26.3%)	4 (21.1%)	
Area of residency	North of Riyadh	162 (70.1%)	50 (21.6%)	19 (8.2%)	0.075	155 (67.1%)	58 (25.1%)	18 (7.8%)	0.968
	South of Riyadh	43 (86.0%)	4 (8.0%)	3 (6.0%)		32 (64.0%)	13 (26.0%)	5 (10.0%)	
	Eastern of Riyadh	130 (80.2%)	26 (16.0%)	6 (3.7%)		110 (67.9%)	39 (24.1%)	13 (8.0%)	
	Western of Riyadh	79 (80.6%)	16 (16.3%)	3 (3.1%)		72 (73.5%)	19 (19.4%)	7 (7.1%)	
	Middle of Riyadh	48 (84.2%)	6 (10.5%)	3 (5.3%)		39 (68.4%)	13 (22.8%)	5 (8.8%)	
Educational level	Illiterate	33 (70.2%)	12 (25.5%)	2 (4.3%)	0.927	24 (51.1%)	16 (34.0%)	7 (14.9%)	0.044
	Primary school	5 (83.3%)	1 (16.7%)	0 (0.0%)		3 (50.0%)	1 (16.7%)	2 (33.3%)	
	Middle school	5 (100.0%)	0 (0.0%)	0 (0.0%)		2 (40.0%)	2 (40.0%)	1 (20.0%)	
	High school	132 (77.2%)	29 (17.0%)	10 (5.8%)		124 (72.5%)	35 (20.5%)	12 (7.0%)	
	Graduate/diploma	144 (78.7%)	30 (16.4%)	9 (4.9%)		123 (67.2%)	44 (24.0%)	16 (8.7%)	
	Post-graduate	143 (76.9%)	30 (16.1%)	13 (7.0%)		132 (71.0%)	44 (23.7%)	10 (5.4%)	
Do you have any missing teeth?	No	243 (74.8%)	61 (18.8%)	21 (6.5%)	0.288	219 (67.4%)	81 (24.9%)	25 (7.7%)	0.739
	Yes	219 (80.2%)	41 (15.0%)	13 (4.8%)		189 (69.2%)	61 (22.3%)	23 (8.4%)	
Have you ever replaced your missing teeth?	No	363 (77.4%)	80 (17.1%)	26 (5.5%)	0.941	320 (68.2%)	112 (23.9%)	37 (7.9%)	0.976
	Yes	99 (76.7%)	22 (17.1%)	8 (6.2%)		88 (68.2%)	30 (23.3%)	11 (8.5%)	
Do you think the replacement of missing teeth is important?	Not important at all	5 (45.5%)	4 (36.4%)	2 (18.2%)	0.100	1 (9.1%)	5 (45.5%)	5 (45.5%)	<0.001
	Somewhat important	131 (77.1%)	30 (17.6%)	9 (5.3%)		109 (64.1%)	45 (26.5%)	16 (9.4%)	
	Very important	326 (78.2%)	68 (16.3%)	23 (5.5%)		298 (71.5%)	92 (22.1%)	27 (6.5%)	

Concerning social media, we did not find any significant differences between demographic groups in terms of agreement with the importance of social media in the selection of dental implant specialists (Table 6).

**Table 6:** Factors associated with participants’ acceptance of the importance of social media in the selection of dental specialists for dental implants.

Parameter	Category	Social media			
		Agree or strongly agree, N = 346	Neutral, N = 170	Disagree or strongly disagree, N = 82	p-value
Gender	Male	171 (55.0%)	99 (31.8%)	41 (13.2%)	0.155
	Female	175 (61.0%)	71 (24.7%)	41 (14.3%)	
Age	20–29	180 (61.6%)	84 (28.8%)	28 (9.6%)	0.070
	30–39	75 (58.1%)	32 (24.8%)	22 (17.1%)	
	40–49	51 (54.3%)	30 (31.9%)	13 (13.8%)	
	50–59	28 (50.9%)	14 (25.5%)	13 (23.6%)	
	>60	12 (42.9%)	10 (35.7%)	6 (21.4%)	
Monthly income	<5000	158 (59.2%)	83 (31.1%)	26 (9.7%)	0.150
	5000–10,000	68 (60.7%)	29 (25.9%)	15 (13.4%)	
	10,000–20,000	60 (51.7%)	34 (29.3%)	22 (19.0%)	
	20,000–30,000	33 (58.9%)	13 (23.2%)	10 (17.9%)	
	30,000–40,000	19 (67.9%)	4 (14.3%)	5 (17.9%)	
	>40,000	8 (42.1%)	7 (36.8%)	4 (21.1%)	
Area of residency	North of Riyadh	131 (56.7%)	66 (28.6%)	34 (14.7%)	0.985
	South of Riyadh	29 (58.0%)	16 (32.0%)	5 (10.0%)	
	Eastern of Riyadh	99 (61.1%)	43 (26.5%)	20 (12.3%)	
	Western of Riyadh	54 (55.1%)	29 (29.6%)	15 (15.3%)	
	Middle of Riyadh	33 (57.9%)	16 (28.1%)	8 (14.0%)	
Educational level	Illiterate	25 (53.2%)	16 (34.0%)	6 (12.8%)	0.546
	Primary school	5 (83.3%)	1 (16.7%)	0 (0.0%)	
	Middle school	3 (60.0%)	1 (20.0%)	1 (20.0%)	
	High school	101 (59.1%)	50 (29.2%)	20 (11.7%)	
	Graduate/diploma	102 (55.7%)	59 (32.2%)	22 (12.0%)	
	Post-graduate	110 (59.1%)	43 (23.1%)	33 (17.7%)	
Do you have any missing teeth?	No	192 (59.1%)	91 (28.0%)	42 (12.9%)	0.771
	Yes	154 (56.4%)	79 (28.9%)	40 (14.7%)	
Have you ever replaced your missing teeth?	No	277 (59.1%)	132 (28.1%)	60 (12.8%)	0.394
	Yes	69 (53.5%)	38 (29.5%)	22 (17.1%)	
Do you think the replacement of missing teeth is important?	Not important at all	4 (36.4%)	3 (27.3%)	4 (36.4%)	0.096
	Somewhat important	103 (60.6%)	51 (30.0%)	16 (9.4%)	
	Very important	239 (57.3%)	116 (27.8%)	62 (14.9%)	

## Discussion

The connection between the prosthodontic and surgical specialties is a close one and cannot be separated. Strong supporting periodontal or peri-implant tissues serve as a sound basis for reliable prosthetic treatment. Moreover, restoring stable periodontal conditions requires appropriate contact types, occlusal schemes, and high-quality prostheses. Clear and effective communication is critical between the surgeon and the prosthodontists throughout the entire treatment process, from planning to maintenance, as these specialties strive towards the same objective of achieving a pleasing aesthetic appearance with a coordinated stomatognathic system [11].

Describing implant failure is simpler than describing implant success or survival, as there could be many reasons behind it, such as clinician experience, systematic condition, parafunctional habits, and smoking status [12,13]. The probability of unloaded implant failure was significantly influenced by the experience of the surgeon, as discussed by Preiskel and Tsolka [14]. Although changes in equipment may have some impact, following a consistent surgical routine is crucial for successful outcomes. Even the best equipment can fail if it is placed by someone lacking surgical expertise. Instead of focusing too much on equipment factors, it is more important to prioritize developing and improving surgical and prosthodontic skills [15]. Melo et al. examined the success rates of dental implants when they are placed by oral and maxillofacial residents and whether the level of training of these residents affects the outcome of the implant treatment. The authors illustrated that when comparing the level of training, the survival rates did not show any significant statistical difference [16]. Generally, patients are motivated to undergo implant replacement for two primary reasons: first, to address functional issues with conventional restorations, and second, to increase their confidence [17]. Chrcanovic et al. evaluated how different factors impact the rate of dental implant failure, with particular attention to the placement of implants by various dental surgeons. The study found that there are varying rates of dental implant failure among different surgeons, which could be significant. While a direct cause cannot be determined, the results suggest that the poor technique, skills, or judgment of some surgeons may have a negative impact on the success of dental implants [18]. Our study showed that 32.1% of participants preferred a periodontist for the placement of dental implants. However, 72.6% of the participants suggested that the implant specialist should have experience in both prosthodontics and surgical procedures for dental implants. The study by Shah et al. showed that while dental specialists who work with implants seem to have a good amount of knowledge about implant-abutment connection and platform switching, there is a lack of understanding when it comes to the mechanical, biological, and technical aspects of the procedure [19].

A previous study by Alqahtani et al. showed that the source of information regarding dental implants among the participants was mainly self-education (46%) [1]. However, our study shows that 44% of participants reported that their main source of information about dental implants was their dentist. In addition, a study by Salim et al. showed that 52.2% of the participants did not know how long the implant would survive, 51.3% of the participants described the dental implants as a screw, and 35.4% suggested that the implants are anchored into the jawbone [2]. On the other hand, in our study, 38.1% of the participants believed that the dental implant lasts for a lifetime, and most of the participants also described the dental implant as a screw (69.6%); 59.7% of the participants also suggested that the implants are anchored into the jawbone. The study by Sharma et al. showed that 54.6% of the participants considered themselves moderately well-informed regarding dental implants, and 31.9% thought that the implant type and material were the most important factors for success. Moreover, 32.6% thought that the initial set-up cost required to incorporate implant surgery into the practice was $2000-$3000 [3]. In correlation, our study demonstrated that 218 of the participants had a very good level of knowledge regarding dental implants, and 24.2% believed that the dental implant cost should be around $500-$1000. As reported by Jha et al., long-term treatment time was the most common limitation in choosing dental implants as a treatment option (57%) [4,5]. However, higher cost (75.4%) was the main barrier to undergoing implant therapy in our study; this also shows a different result compared to the study by Simensen et al., which showed that cost is less important among patients seeking implant therapy [20]. In our study, some of the participants disagreed that social media could influence the choice of an implant specialist, which interestingly explains that social media platforms are not always validated tools for advertising dental implants.

## Conclusions

In this study, different factors were related to choosing an implant specialist among the population. In our study, the most frequently cited factor was dental implant cost, followed by dentist qualification. Moreover, the knowledge regarding dental implants was average among most of the studied groups, so we recommend increasing knowledge and awareness regarding the complications as well as the possible outcomes of dental implants.
